# Finite Element Analysis and Experimental Investigation of Cut Surface Formation of Magnetic Silicon Steel in Shear Cutting

**DOI:** 10.3390/ma14216415

**Published:** 2021-10-26

**Authors:** Łukasz Bohdal, Agnieszka Kułakowska, Marcin Kułakowski

**Affiliations:** Department of Mechanical Engineering, Koszalin University of Technology, Racławicka, 75-620 Koszalin, Poland; agnieszka.kulakowska@tu.koszalin.pl (A.K.); Marcinkulakowski@wp.pl (M.K.)

**Keywords:** grain-oriented silicon steel cutting, shearing mechanism, parametric study, cut surface analysis

## Abstract

Shear cutting allows for shaping materials with any length of cutting line with high efficiency and without negative thermal effects, but it causes stresses and deformations in the cutting zone of the material. This has a negative effect on the magnetic properties of the sheet in the areas of the cut edge. The main problem on production lines is to ensure appropriate control of the process so as to obtain the appropriate technological quality of the cut edge, free of not only defects in the form of burrs and shape deviations, but also the minimum deformed zone. This task is difficult due to the large number of control variables, the influence of which on the shaping of the material and the formation of the cut edge is not fully understood. The article attempts to determine the course of the cutting process and to examine the influence of control variables on the formation of the cut edge in the shear-slitting process in which the tools perform a rotary motion. For this purpose, FEM modeling, vision techniques and experimental studies were used. A 3D model of the process was developed, which enables a detailed analysis of the states of stresses, strains, displacements and fracture mechanisms of the material. The simulation results were verified using vision techniques, which were used in the work to observe the flow and cracking mechanisms of the material. Parametric analyses were performed for the process control variables. The research showed a significant influence of the cutting velocity and the clearance between the tools on the formation of the cut edge. The most homogeneous surface of the cut edge with the minimum burr height was obtained for the following parameters: rake angle *α* = 15–30°, horizontal clearance *h_c_* = 0.03 mm and slitting velocity *v*_2_ = 15 m/min. The developed results can be useful for controlling the cutting process on production lines in terms of maximum process efficiency while maintaining the appropriate technological quality of the cut edge.

## 1. Introduction

Magnetic materials, which include, among others nanocrystalline and amorphous strips, grain oriented or non-oriented electrical silicon steels should be cut to specific dimensions to obtain final products, because they are produced in large rolls. Often times, laser, guillotine, shear-slitting or punching techniques are used to cut the desired shapes. Each of these processes causes unfavorable physical phenomena in the shaped product, which can be significantly reduced by applying appropriate guidelines. In the case of shear cutting, these are large plastic deformations in the cutting zone, high residual stresses, formation of burrs, excessive fracture zone and edge cracks. In the case of laser cutting, there is also the disadvantage of hot ablation phenomena. In the shaping of magnetic steels, it is very important to ensure not only the appropriate geometry of the cut edge, but also the preservation of the magnetic properties given during the manufacturing processes. The most negative effects of the cutting process on magnetic properties include a change in the distribution of flux density, hysteresis loss, changes in the maximum value of magnetic induction, changes in the width of the hysteresis loop and average residual magnetization and changes in hysteresis loop surface area. In many studies, the authors try to determine the mechanisms of the formation of stresses and deformations in the sheet by means of experimental tests, which is difficult, especially in the case of invasive methods which do not always ensure adequate accuracy and cannot be used for the analysis of thin sheets and strips.

Currently, the knowledge about the shear-slitting of magnetic materials is limited. The authors focus mainly on research on structural steels and aluminum alloys where the magnetic properties are not important. Meehan and Burns [1] investigated the influence of cutting tools geometry and they edge sharpness on instabilities of polyethylene terephthalate web (PET thermoplastic). Ma et al. [2] analyzed the influence of selected process parameters on burr formation during cutting aluminum alloy. Lu et al. [3,4,5] developed a basis of shear-slitting which allowed to burr reduction at aluminum sheet cut edge. It has been shown that there is a critical clearance value between the tools (horizontal clearance), the exceeding of which causes an increase in the burr height above the permissible value on industrial lines. Vertical clearance, which influenced the wear of the cutting tools, also played an important role in the process. By creating local tension/shear in material, the authors made it easier to separate the material early and reduce burrs.

In study [6], the process of shaping the AA6014 aluminum alloy was investigated by means of sensitivity analyses, which consisted in the use of various variants of control parameters. As a result, optimal settings were identified to minimize burrs and slivers. In trimming cutting processes, it is possible to reduce the stress values in the cutting zone by sheet supporting [7]. The paper in [7] also discusses the effect of trimming clearance on the cut edge formation. Hubert et al. [8] used numerical modeling to analyze the rolling and edge trimming process. As a result of the analyses, it was possible to forecast stress fields in the cut edge. In the work [9] strength tests were carried out to analyze the mechanisms of neck formation and fracture. The test samples were previously cut out and had microcracks. This allowed for study of the influence of microcracks on the cut surface on the strength properties of the aluminum alloy. Pluta et al. [10] analyzed the magnetic properties of sheets cut into strips. The focus was on silicon sheets with different permeability and silicon content.

In the area of magnetic materials, the effect of slitting grain-oriented electrical core steel on iron loss was investigated in study [11]. Godec [12] analyzed the influence of slitting on selected magnetic properties of grain-oriented electrical steel. Sheets of different widths were used (from 40 to 660 mm). It was found that on the constructed stand the main problem related to the deterioration of magnetic properties was the formation of deformations due to the use of inappropriate tools.

In the literature, it is possible to find items related to FEM modeling of the shear-slitting process, but their number is negligible. This is due to the need to build complex 3D models that take into account the rotation of tools and sheet metal movements, which is difficult, time consuming and not possible to model in a 2D system. It is also necessary to create a physical and mathematical model taking into account geometric and physical nonlinearities and advanced constitutive relationships describing nonlinear phases of the process, such as plastic flow of material and cracking. The work [13] shows the possible applications of an updated description of Lagrange and Lagrange Euler (ALE) formulations to simulate the formation of construction steel cut edge at a typical incremental step. The developed 2D and 3D models made it possible to analyze the deformations, states of stresses and displacements in the steady phase of the process and to simulate defects arising after cutting, e.g., sheet bends, depending on the adopted technological parameters (the geometry of the upper knife and the clearance between the knives). The elastic-plastic material model was used with the implemented Oyane fracture criterion, which did not take into account the strain rate and the influence of temperature on the plasticizing stresses of the material. The authors of the work [14] developed 3D simulation models of the process of shear-slitting aluminum alloys. To describe the material properties, an elastic-plastic model with implemented the Cockroft and Latham fracture criterion was used. Due to the complexity of calculations, the simulations did not allow the observation of cut surface defects after complete separation of the sheet metal.

In the present work, the main aim is to analyze the effect of shear slitting process technological parameters on formability of cut surface of grain-oriented silicon steel through FEM simulation and experimental research. Due to the complexity of the process, it was considered an initial and boundary value problem. The models took into account physical, geometric and thermal nonlinearity, assuming partial knowledge of the boundary conditions in shearing zone. Developed mathematical and physical models allow for analysis of the cut edge forming process. Not only the steady phase of the process was taken into account, but also the unsteady with plastic flow and cracking phenomena. Using the developed models, it is possible to forecast deformation and stress states at any time during the process and at any point in the sheet. To describe nonlinear phenomena on a typical incremental step, the updated Lagrange description with a stepwise and co-rotational coordinate system was used. The strain and strain rate states are nonlinear dependences without any linearization. In the experimental tests, a specially designed test stand was used, enabling the use of high cutting speeds and variable settings for horizontal and vertical clearance. As a result, it was possible to validate the results of numerical simulations and observe the cutting-edge forming process. In order to observe the separation process in detail, vision systems based on the digital image correlation (DiC) method were used.

## 2. Shear Cutting Process

**Figure 1:**
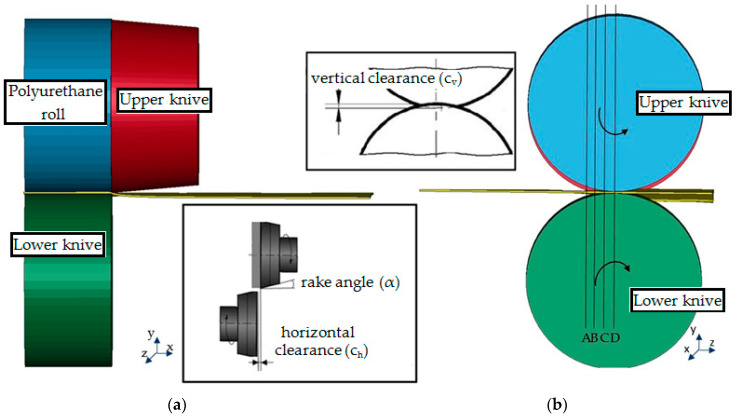
Illustration of the shear-slitting process with specifying parameters: (**a**) Front view; (**b**) Side view.

Shear cutting is the process of shaping metal sheets by separating one part of the material from another. Such separation is accompanied by large plastic deformations, which lead to the deterioration of the cohesion of the material. There are different cutting methods depending on the type of material as well as the shape of the workpiece. One popular method of shear cutting is the shear-slitting process in which the tools rotate (Figure 1). Industries use shear-slitting to split electrical steel (generally produced in coils of 800–1000 mm in width) into several smaller widths.

The sheet is first plastically deformed (A–B). The continued deformation leads to a ductile fracture (B–C) and then complete separation of the parts (D) [1,13]. The material separation process is a process of large deformation with complex physical features.

## 3. Numerical Modeling

**Figure 2:**
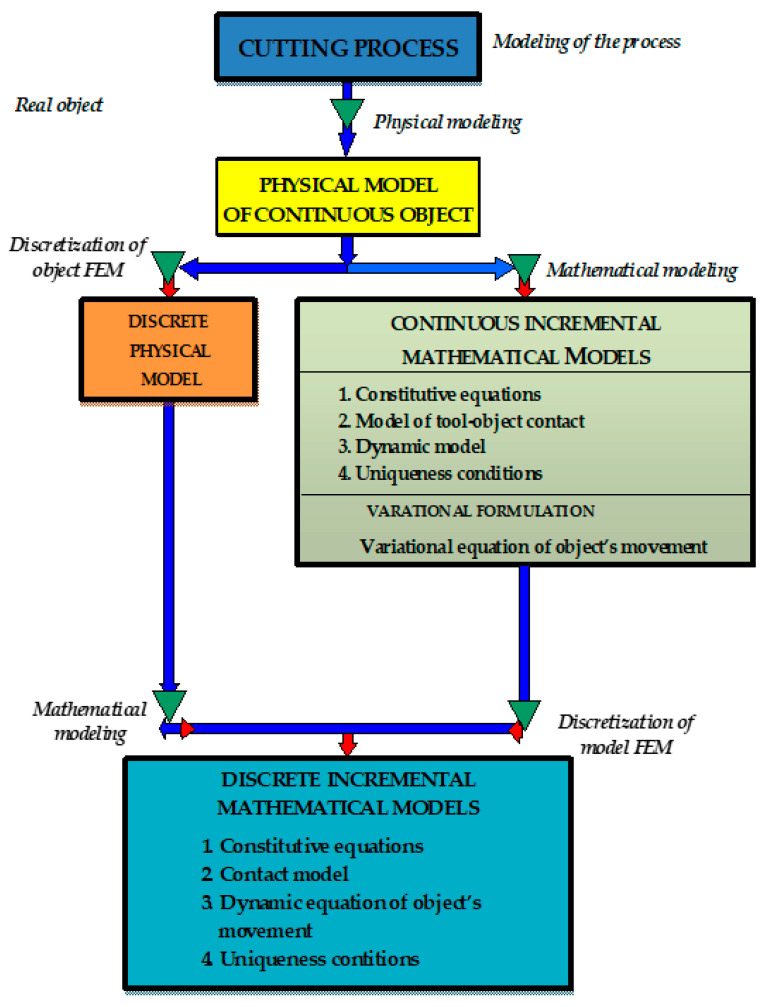
Flow chart of modeling the cutting process.

In order to analyze the physical phenomena occurring during the cutting process of magnetic materials, mathematical and physical modeling was carried out with the use of incremental models, taking into account the history of strains and strain rates. Physical modeling of the real object allowed for the development of a physical model with assumptions and simplifications (Figure 2).

With the help of mathematical modeling, continuous, incremental mathematical models were developed including the model of contact tools with the sheet, constitutive equations, uniqueness conditions and dynamic equation of motion. The variational equation of motion of the object was obtained as a result of the variational formulation. The construction of a discrete physical model was performed by means of discretization of the object with finite elements.

Discrete, incremental mathematical models of the physical model were obtained using the approximation of the continuous mathematical model using the finite element method or the mathematical modeling of a discrete physical model. Building a computer model in the FEM environment included conducting model sensitivity analyses to changes in the geometry of finite elements and mesh refinement. The final step was to prepare the data for calculations, select a solver, calculate and edit the results.

### 3.1. Basic Relationships

When modeling the behavior of the material under high loads, the effects of thermo-elasticity and thermo-viscoplasticity in the reversible zone and in the non-reversible zone were taken into account. The formulation of the model assumes that the typical small incremental components $\Delta \varepsilon_{ij}$ of the strain tensor $T_{\Delta \varepsilon }$ can be expressed as the sum of the thermo-elastic $\Delta \varepsilon_{ij}^{\left(TE\right)}$, viscoplastic $\Delta \varepsilon_{ij}^{\left(VP\right)}$ and thermal $\Delta \varepsilon_{ij}^{\left(TH\right)}$ incremental strains:

(1) $$\Delta \varepsilon_{ij}^{}=\Delta \varepsilon_{ij}^{\left(TE\right)}+\Delta \varepsilon_{ij}^{\left(VP\right)}+\Delta \varepsilon_{ij}^{\left(TH\right)},$$

The constitutive law for thermo-elastic material with an isotropic properties and temperature-dependent module can be expressed as:

(2) $$\Delta \sigma_{ij}^{}=C_{ijkl}^{\left(TE\right)}\cdot \left(\Delta \varepsilon_{kl}-\Delta \varepsilon_{kl}^{\left(VP\right)}-\Delta \varepsilon_{kl}^{\left(TH\right)}\right)+\Delta C_{ijkl}^{\left(TE\right)}\cdot \varepsilon_{kl}^{\left(E\right)},$$

where $\Delta \sigma_{ij}^{}$ are the incremental components of the second Piola-Kirchhoff stress, $\varepsilon_{kl}^{\left(E\right)}$ are the accumulated components of the elastic strain tensor at time *t* [15]. The component of the temperature-dependent elastic constitutive tensor can be expressed as:

(3) $$\;C_{ijkl}^{\left(TE\right)}\left(T\right)=\lambda \left(T\right)\cdot \delta_{ij}\cdot \delta_{kl}+\mu \left(T\right)\cdot \left(\delta_{ik}\cdot \delta_{jl}+\delta_{il}\cdot \delta_{jk}\right),$$

where $\lambda \left(T\right)=\frac{E\left(T\right)\cdot \nu \left(T\right)}{\left[1+\nu \left(T\right)\right]\cdot \left[1-2\nu \left(T\right)\right]},µ\left(T\right)=\frac{E\left(T\right)}{2\left[1+\nu \left(T\right)\right]},\;\delta_{ij}\;\mathrm{is}\;\mathrm{the}\;\mathrm{Kronecker}\;\mathrm{delta},\;E\left(T\right)$ is the temperature-dependent Young modulus, ν(T) is the temperature-dependent Poisson ratio.

Its increments are calculated from the equation:

(4) $$\;\Delta C_{ijkl}^{\left(TE\right)}\left(\Delta T\right)=\frac{\partial C_{ijkl}^{\left(TE\right)}\left(T\right)}{\partial T}\Delta T,$$

From the equation:

(5) $$\Delta \varepsilon_{ij}^{\left(TH\right)}≅\alpha_{m}\left(T\right)\cdot \Delta T\cdot \delta_{ij},$$

the components of the thermal increment strain tensor are calculated, where $\alpha_{m}$ is the coefficient of thermal expansion [16,17]. The constitutive equation of mixed hardening for an isotropic material is as follows:

(6) $$\Delta \sigma_{ij}^{}=C_{ijkl}^{\left(TE\right)}\cdot \left(1-{\tilde{S}}^{**}\right)\cdot \;\Delta \varepsilon_{kl}-C_{ijkl}^{\left(TE\right)}\cdot \left(\Delta \varepsilon_{kl}^{**}+\alpha_{m}\cdot \Delta T\cdot \delta_{kl}\right)+\Delta C_{ijkl}^{\left(TE\right)}\cdot \varepsilon_{kl}^{\left(E\right)}=C_{ijkl}^{\left(TE\right)*}\cdot \;\Delta \varepsilon_{kl}+\Delta \sigma_{ij}^{**},$$

Formula includes the combined effects of thermo-elasticity and thermo-viscoplasticity, where:

(7) $$C_{ijkl}^{\left(TE\right)*}=C_{ijkl}^{\left(TE\right)}\left(1-{\tilde{S}}^{**}\right),\Delta \sigma_{ij}^{**}=-C_{ijkl}^{\left(TE\right)}\cdot \left(\Delta \varepsilon_{kl}^{**}+\alpha_{m}\cdot \Delta T\cdot \delta_{kl}\right)+\Delta C_{ijkl}^{\left(TE\right)}\cdot \varepsilon_{kl}^{\left(E\right)}$$

The constitutive model (6) is used for practical engineering analysis, in this case, for the shear slitting process analysis.

### 3.2. FE Model

**Figure 3:**
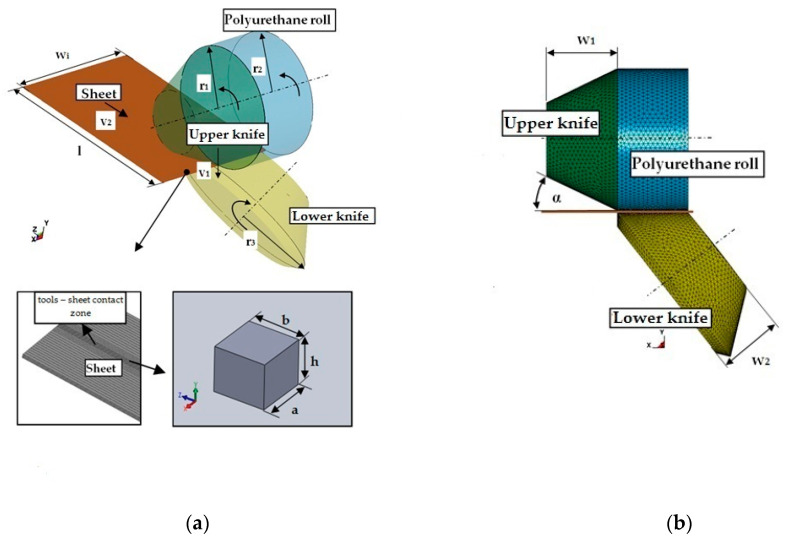
FEM simulation model of the shear-slitting process: (**a**) Isometric view with discretization of sheets scheme; (**b**) Front view.

The current amount of knowledge in the literature is limited in terms of the results of research on the influence of technological parameters and conditions of cutting processes on the cut edge forming of electrotechnical materials. An important problem is to determine the width of the resulting stress and deformation zone in the material along the cutting line, depending on the adopted parameter values and their relationship with the quality of the cut edge. The models developed enable a comprehensive and non-invasive analysis of the influence of process conditions and technological parameters on the states of stress, deformation, displacement and the quality of the cut edge. In order to analyze the states of stresses, strains and displacements in detail, it was necessary to build a three-dimensional model taking into account the 3D state of strain and 3D state of stress and the actual boundary conditions of the process (Figure 3).

The simulations took into account very important parameters neglected in many studies, such as value of the rake angle (*α*), knives radius values, length of the cutting line and method of supporting the sheet. Two phases of the process were modeled in the work in the form of setting the overlap (vertical clearance value) of the knives with the speed *v*_1_ = 200 mm/s and then the phase of longitudinal movement of the sheet with the speed *v*_2_ performed by the rotation of the shears (Figure 3). The shear slitting process takes place under a constant temperature (ΔT=0). Moreover: λ(T)=λ, ρ(T)=ρ, $\Delta \varepsilon_{ij}^{\left(TH\right)}=0$, $\;\Delta C_{ijkl}^{\left(TE\right)}\left(\Delta T\right)=0$,$\;C_{ijkl}^{\left(TE\right)}=C_{ijkl}^{\left(E\right)}$. ET 122-30 with thickness of *t* = 0.3 mm grain-oriented silicon steel was used in study (Table 1). Typical chemical composition of the ET steels consists of Si (2.50–4% weight), Mn (0.03–0.15%), Cu (0.03–0.3%) and P (0.02–0.2%), the balance being Fe.

The 8-node SOLID 164 element type with hourglass control and reduced integration was used for discretization. This allowed to take into account in the simulations not only the possibility of movement along the x, y and z axes, but also the rotation of finite elements around the whole axis. In order to reduce the calculation time, sheet areas with different degrees of mesh densities and tools treated as non-deformable bodies, i.e., *E* → ∞ were accepted. To eliminate the influence of non-uniform mesh on the final results, the sheet has been divided into cubic elements. Coulomb’s friction model was used for the description of friction conditions in contact areas according to the formula:

(8) $$\;\mu_{c}=F_{D}+\left(F_{s}\;-\;F_{D}\right)e^{-D_{c}\left|v_{rel}\right|},$$

where *F_S_* and *F_D_* are static and dynamic friction coefficients, respectively, *D_C_* is the exponential decay coefficient, *v_rel_* is relative velocity of the surfaces in contact and *e* is Euler’s number. To determine the influence of yield stress on plastic deformation, taking into account the damage of the material, the Johnson-Cook constitutive equation additionally considers the impact of strain rate and temperature on the yield stress values used [18,19,20,21]:

(9) $$\sigma_{Y}=\left(A+B\cdot \varepsilon^{n}\right)\left(1+C\cdot ln{\dot{\varepsilon }}^{*}\right)\left[1-{\left(\frac{T-T_{r}}{T_{m}-T_{r}}\right)}^{m}\right],$$

where *A* is the yield strength of the material, *B* is the strain hardening constant, *C* is the strain rate strengthening coefficient, *σ_Y_* is the yield stress, *ε* is the equivalent plastic strain, ${\dot{\varepsilon }}^{*}\;$is the normalized effective plastic strain rate, *m* is the thermal softening coefficient, *n* is the strain hardening coefficient, *T_m_* is the material melting temperature, *T_r_* is the room temperature [21]. For ET 122-30 steel: *A* = 104.3 MPa, *B* = 445.6 MPa, *C* = 0.041 [-], *m* = 0.54 [-], *n* = 0.46 [-] [22].

### 3.3. Sensitivity Analysis

**Figure 4:**
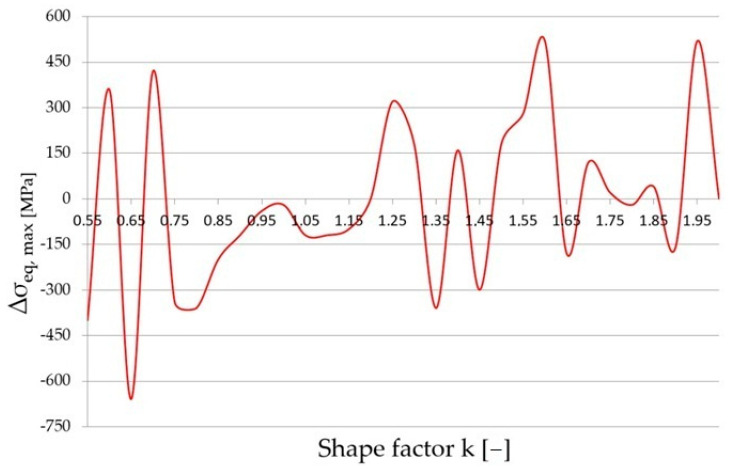
The influence of shape factor *k* on increment of equivalent stress.

In processes with large deformation and material separation phenomena, it is necessary to ensure minimum prediction error of slitting variables and minimal simulation cost [23]. To determine the optimal parameters of the solver a series of numerical simulations are carried out. The computer simulations were performed for different shapes and quantities of finite elements used in the shear slitting model. A total of 30 simulations are carried out for different shape factor where *k = h/a* = 0.55 ÷ 1.95 in the tool sheet contact zone (Figure 3a), while dimension *b* is treated as a constant. Example results are given in Figure 4. The linear characteristics of $\Delta \sigma_{eq{,}_{max}}$ between *k =* 0.95 ÷ 1.2 and *k =* 1.75 ÷ 1.85 and values close to 0 can be observed. For the different values of shape factors, results could not be accurate; $\Delta \sigma_{eq{,}_{max}}\;$is defined as increment of the von Mises equivalent stress (the difference between the maximum equivalent stresses for the current and previous shape factor *k*). Taking into account accuracy of crack propagation the most optimal solution is to use shape factor k≅1. Finally, the model features a very fine mesh with size of 0.015 mm and shape factor *k* = 1 in the tool sheet contact zone, which is required to accurately capture the crack path and high strain gradient associated with the shear slitting process. As such phenomena do not occur in regions outside of the tool sheet contact zone (Figure 3a), these were modeled with larger element sizes in order to reduce the calculation times.

## 4. Experimental Validation

### 4.1. Experimental Setup

**Figure 5:**
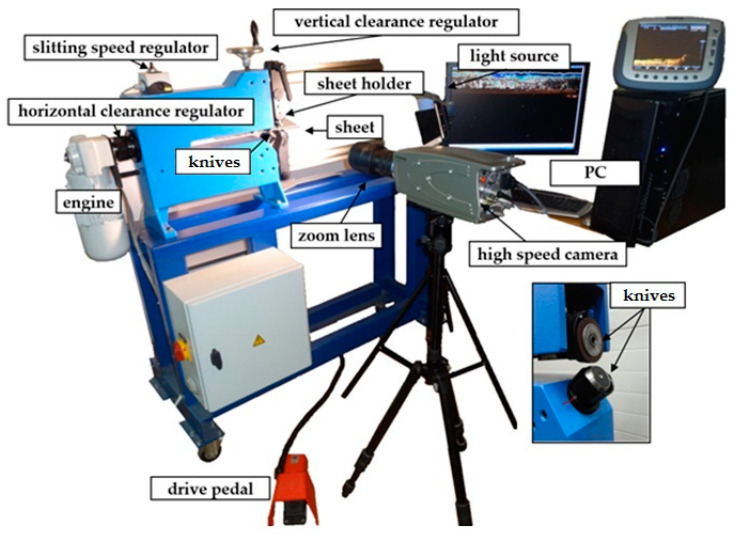
Experimental Setup.

In order to conduct experimental studies to validate the FEM models and to explain the influence of the cutting process conditions on the geometric structure of the cut edge, a special stand was used, consisting of rotary shears type KSE 10/10, located in the Department of Technical Mechanics and Strength of Materials at the Koszalin University of Technology, were used for the tests (Figure 5). The device is used for cutting strips, discs and rings from sheet metal sheets on industrial production lines. It provides very high efficiency and high quality of the cut products. It is equipped with elements for internal and external flanging on cut discs and rings. In addition, the device is equipped with stainless guides and an exchangeable clamping plate. The machine is driven in one, two or stepless stages through a motor with a gear and a brake. The engine power is 0.37 kW. The diameter of the discs and rings to be cut is set using a scale. The sheet metal can be fixed pneumatically or hydraulically. The drive is set to the upper knife. The clearance is adjusted via a threaded socket with a scale. It is possible to use a special needle if it is necessary to cut out elements of an oval shape. A polyurethane roll is used to allow the sheet to be moved in horizontal direction under non-sliding contact conditions (where: *r*_1_ = *r*_2_ = 15 mm, *r*_3_ = 20 mm, *l* = 80 mm, *w_i_* = 40 mm).

Due to the high complexity and speed of the course of physical phenomena, an advanced vision method (digital image correlation) was used in the cutting process in the form of a specially designed monitoring system, which consisted of a high-speed camera i-SPEED TR, zoom lens, light sources and the GOM Correlate and i-SPEED Suite software. The resolution of 1280 × 1024 pixels, and at the speed 2000 fr/s were used in image registration.

### 4.2. Design of Experiments

In order to determine the important technological parameters affecting the course of the process and the quality of the cut edge, preliminary tests were carried out. As a result of the research, the input, output, constants and disturbing factors were determined. The input factors include: horizontal clearance (*c_h_*), slitting velocity (*v*_2_) and a rake angle of the upper knife (*α*). The ranges of the variability of the studied factors are summarized in Table 2.

## 5. Results and Discussion

### 5.1. Generation of Slit Edges

**Figure 6:**
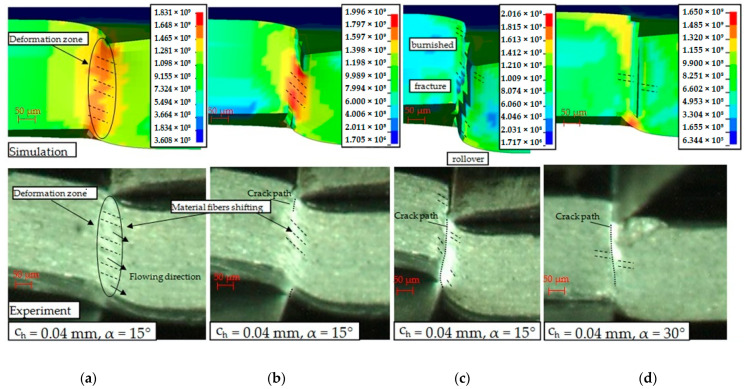
Formation of slit edges at sheet cross section: (**a**) plastic flow phase; (**b**) cracking phase; (**c**) final separation; (**d**) cracking phase with different tool geometry.

The mechanical cutting process consists of an elastic phase in which the tools press against the surface of the sheet, elastoplastic in which the material strengthens as a result of a significant increase in stresses and deformations in the cutting zone, elastoplastic in which damage occurs in which the first cracks appear at the area of contact of the tool blades with material and the fracture phase in which the material separates completely. As mentioned, the proposed method with the use of a vision system allowed for the observation and analysis of the phenomena of material deformation and its flow, followed by cracking and analysis of its trajectory. A number of tests have been carried out to ensure proper image sharpness and adequate illumination of the areas of the cutting zone. Figure 6 shows different phases of process with selected process conditions both for FEM simulations (equivalent stress distribution) and experiments. During the first phase of the process, the cutting tools indent the sheet and create a small zone of elastic deformation near the cutting edges of the tools, which moves deeper into the material as pressure increases. As a result of the increase in stresses in the deformation zone until reaching the yield point, the next phase of the slitting process takes place. In this phase, there is a further increase in the bending moment and the tensile stress values, and the extruding depth of the sheet increases gradually. It is possible to observe the tendency in which the deformation zone is extended on a whole sheet thickness. This is confirmed by the conducted experimental studies and recorded images in which the areas with high light intensity are visible, where the greatest deformations occur (Figure 6a). In the area of the greatest deformations, the formation of characteristic fiber patterns at cross section of the material can be observed. As the advancement of the process increases, the degree of their displacement and mutual shift increases (Figure 6b). As the research shows, the degree of shift also depends on the geometry of the upper knife. The flowing mechanism and area of this phenomena is very similar both for simulation and experimental observations. The fracture process may begin not only in the vicinity of the cutting edges of both shears. It is possible to crack the material in the vicinity of the cutting edge of only one of the tools and its further propagation. In the case shown in Figure 6b, two cracks can be observed, one near the upper edge of the knife and the other near the lower edge of the knife, which propagate towards the thickness of the plate and meet inside the material. The model correctly reflects this phenomenon (Figure 6c).

**Figure 7:**
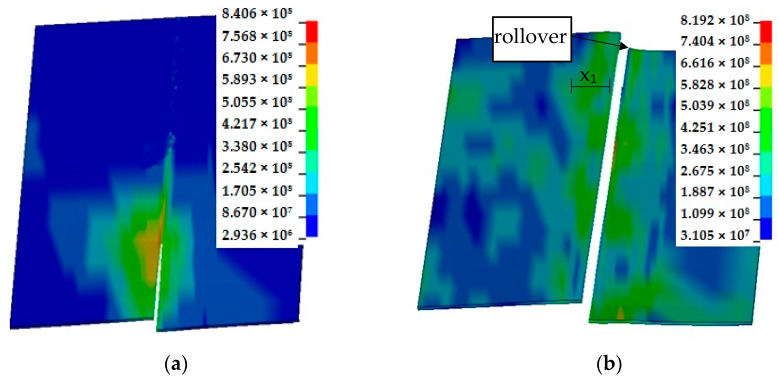
Formation of slit edges along the shearing line: (**a**) 25% of sheet length; (**b**) 100% of sheet length (*c_h_* = 0.04 mm, *α* = 15°).

The developed model allows for simulation of cut surface features, including rollover, burnished and fracture areas. Figure 6d shows cracking phase for changed upper knife geometry when the rake angle is carried out *α* = 30°. The use of such a rake angle configuration resulted in obtaining a gap more perpendicular to the cross section of the sheet than in the case where *α* = 15°. Smaller outflows and reduced rollover were obtained. The cracking process began near the cutting edge of the upper knife. The width of mechanically affected zone and material fibers displacement is low. As the sheet cut, it moves tangentially to the cutting tools. Hence, the tool material contact area is inclined to the horizontal at an angle. The greatest equivalent stresses occur in the vicinity of the cutting edge and decrease as the distance from the edge increases (Figure 7a), while normal compressive stress is split into two components along the *Y* and *Z* axes contributing to the two normal stresses. The bending moment generated during the process has a particular impact on the final stage of the process, resulting in the formation of a rollover of the cut surface at the end of the shearing line (Figure 7b).

### 5.2. Parametric Study

**Figure 8:**
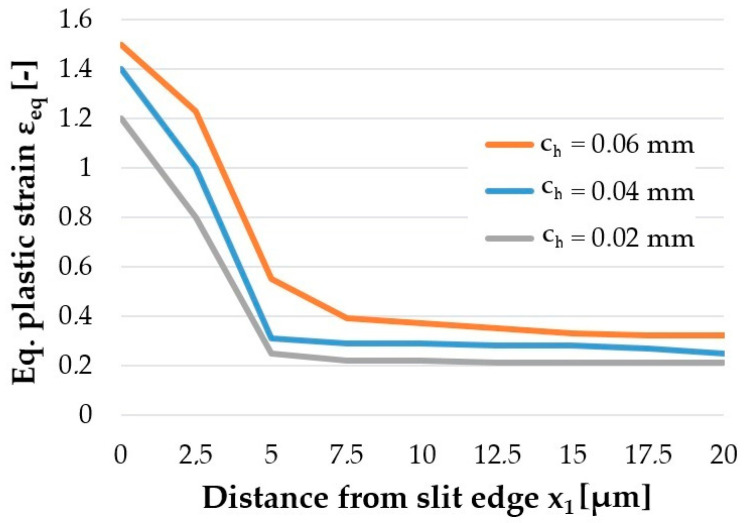
Effect of horizontal clearance *c_h_* on the equivalent plastic strain generated on cut edge.

**Figure 9:**
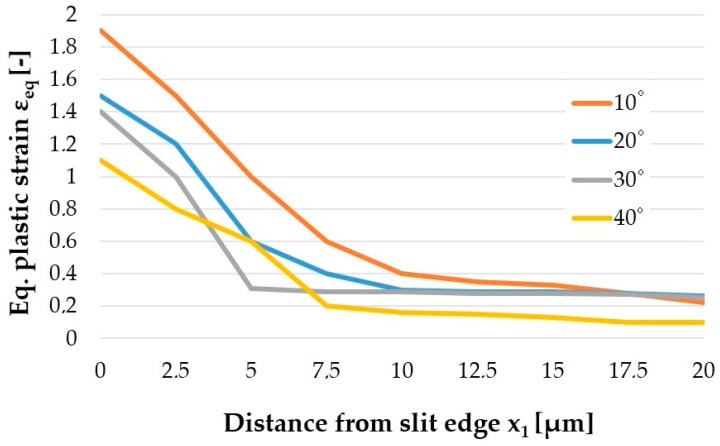
Effect of upper knife rake angle *α* on the equivalent plastic strain generated on cut edge.

**Figure 10:**
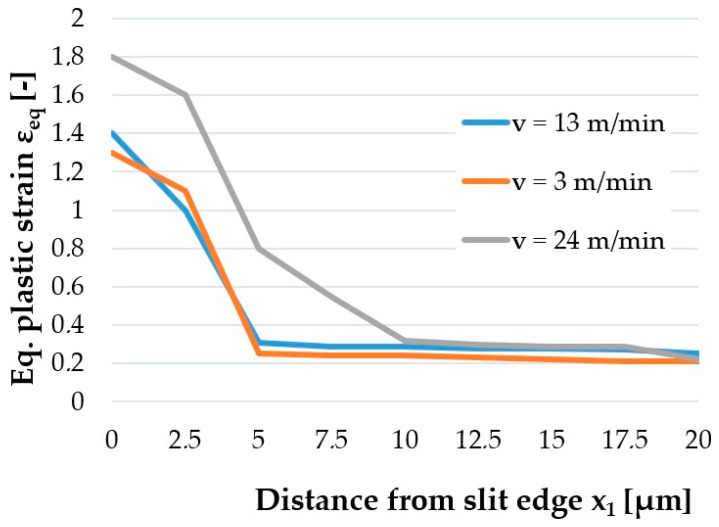
Effect of shear-slitting velocity *v*_2_ on the equivalent plastic strain generated on cut edge.

The quality of the cut edge for construction materials is generally determined by the width of the burnished and fracture zones and the burr height [24]. The work includes additional measurements of significant importance from the operational point of view of electrical sheets, such as measurements of the maximum value of equivalent plastic strain on the cut edge and the extent of the damage around the edge. The influence of studied factors on equivalent plastic strain state at cut surface of sheet is studied and the results are presented in Figure 8, Figure 9 and Figure 10.

**Figure 11:**
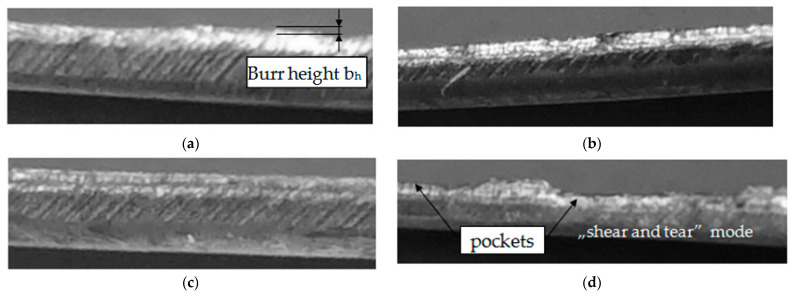
View of the sheared edges under study: (**a**) *v*_2_ = 3 m/min, *c_h_* = 0.04 mm, *α* = 30°; (**b**) *v*_2_ = 13 m/min, *c_h_* = 0.04 mm, *α* = 30°; (**c**) *v*_2_ = 18 m/min, *c_h_* = 0.04 mm, *α* = 30°; (**d**) *v*_2_ = 3 m/min, *c_h_* = 0.08 mm, *α* = 30°.

**Figure 12:**
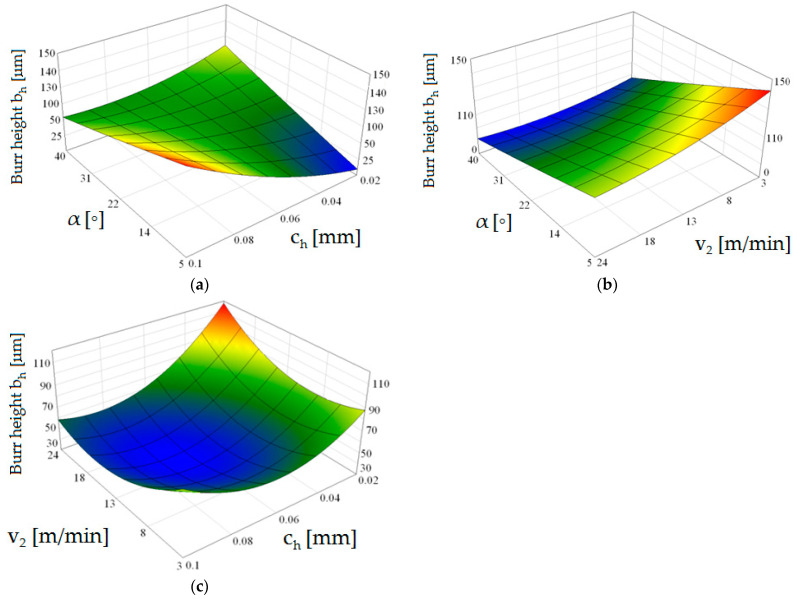
Graphs of the dependence of the burr height on the selected process parameters (**a**–**c**).

**Figure 13:**
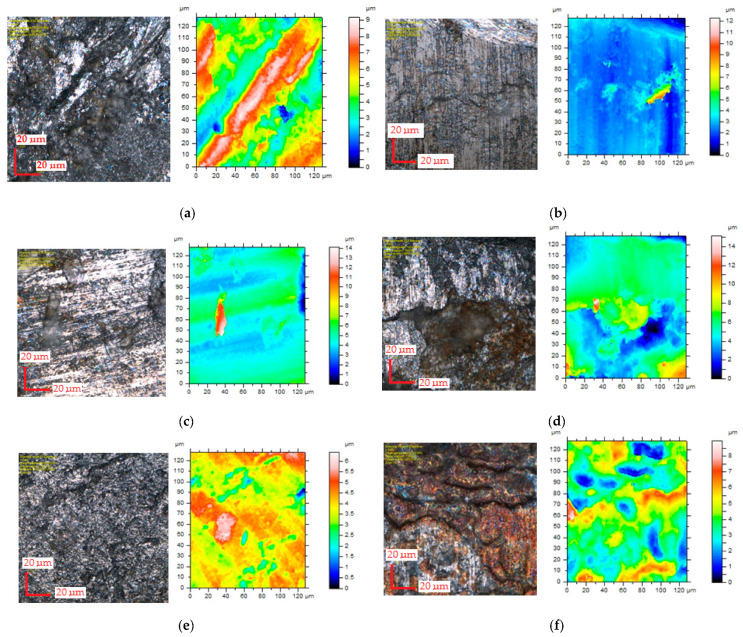
Influence of selected process parameters on contour maps of burnished areas of cut surfaces: (**a**) *v*_2_ = 3 m/min, *c_h_* = 0.04 mm, *α* = 30°; (**b**) *v*_2_ = 13 m/min, *c_h_* = 0.04 mm, *α* = 30°; (**c**) *v*_2_ = 18 m/min, *c_h_* = 0.04 mm, *α* = 30°; (**d**) *v*_2_ = 3 m/min, *c_h_* = 0.08 mm, *α* = 30°; (**e**) *v*_2_ = 13 m/min, *c_h_* = 0.08 mm, *α* = 30°; (**f**) *v*_2_ = 18 m/min, *c_h_* = 0.08 mm, *α* = 30°.

The research showed a significant concentration of the maximum values of plastic strain at the edge in the burnished zone and a gradual decrease in their values with increasing distance from the edge. The maximum equivalent deformation was 1.9 and it took place during cutting with the settings: *c_h_* = 0.04 mm, *α* = 10°, *v*_2_ = 13 m/min. Equivalent plastic strain exponentially decays away from the edge. The values of equivalent strains appear to stabilize at a depth of approximately 10–15 µm from cut edge. The damaged area increases with increasing horizontal clearance (Figure 8). From Figure 9 it can also be seen that the equivalent plastic strain decreases as the rake angle *α* of the upper knife increases. The effect of slitting velocity on equivalent plastic strain state in range of velocities between *v*_2_ = 3–13 m/min is smaller than when high velocities are used (Figure 10). It is observed that higher equivalent plastic strain values and wider mechanically affected zone are achieved for higher slitting velocities. The effect of selected process parameters on burr formation is analysed experimentally and example of results are given in Figure 11, Figure 12 and Figure 13. The values of burr height are measured by using “Vision Engineering” optical microscopy. Examples of cut surfaces are presented in Figure 11.

The burr height depends both on the horizontal clearance value, rake angle and slitting velocity. The most homogeneous state of cut surface and its high quality is obtained using parameter configurations: *α* = 30°, *c_h_* = 0.04 mm and *v*_2_ = 13 m/min (Figure 12a–c).

Similar quality of the cut surface when *α* = 30° and *c_h_* = 0.04 mm can be obtained for the cutting velocity *v*_2_ = 18 m/min. The limit value of horizontal clearance in the correlation of analyzed process parameters is value *c_h_* = 0.06 mm for selected thickness of material (Figure 12a,c). An increase in clearance above this value causes non-uniform burr formation along the cutting line. An unfavorable effect is also the occurrence of areas formed as a result of an excessive concentration of tensile stresses causing the formation of deep pockets if stretching is applied parallel to this surface (Figure 11d). This phenomenon often occurs as a result of inadequate and too high horizontal clearance along the shearing line, which causes separation of burrs from the cut part. As a result of the creation of additional forces in the cutting zone, the burrs are torn off from the side of the sheared surface. With low clearance values in the range of *c_h_* = 0.02–0.04 mm increasing, the rake angle α results in an increase in burr height, while with a higher clearance value (*c_h_* = 0.06–0.08 mm) this trend is reversed (Figure 12a). If the value of rake angle is carried out between *α* = 5–15°, the influence of cutting velocity on the burr height increases (Figure 12b).

Figure 13 shows photographs and contour maps of burnished areas of cut surfaces obtained in the dependence of analysed process parameters. Maps and photographs are made using confocal microscope LEXT OLS4000. On cut surface in macro and micro scales irregularities (asperities) can be seen in which heights and alignment can affect the tribological properties of steel after the cutting process [25,26]. The quality of the burnished area can have a significant impact on the operational properties of machine components trough the friction conditions in the contact areas, the contact stresses, fatigue strength, joint tightness and magnetic properties. The observations show that for low horizontal clearance and cutting velocity, the burnished area consists of matte and glossy areas with a wavy structure (Figure 13a). It can be seen in displacements of adjacent material layers with respect to each other which are arranged at a significant angle to the direction of shearing. An increase in cutting velocity results in an increase in the regularity of the profile which is more glossy and free of significant asperities (Figure 13b,c). Increasing the horizontal clearance to *c_h_* = 0.08 mm using low cutting velocity *v*_2_ = 3 m/min caused the appearance of fracture zones at burnished area (Figure 13d). Increasing the cutting velocity results on changes in distribution and number of fracture zones and furrows on burnished profile (Figure 13e,f), according to results performed by Dzidowski [27] who analyzed cutting mechanisms of iron it can be a result of increasing of local stretching stresses leading to cracking along the shear bands.

In terms of the quality of the cut edge, horizontal clearance *c_h_* has the greatest impact on the formation of product defects. This is mainly because this parameter has a significant impact on the course of the burr formation process, which is a significant problem encountered on production lines. It is then necessary to grind the surface, which changes the state of stresses and strains on the intersection surface and increases the cost of the process. Another factor is the rake angle value which influences the concentration of the maximum plastic deformations in cut edge. If the angle is small, the wear zone is wider, but the tools are subject to less wear. In the case of magnetic steels, it is advisable to make this zone as narrow as possible so as to reduce hysteresis losses in the areas of the cut edge. As our research has shown, it is recommended to use angles around *α* = 15–30°. When cutting other materials, smaller angles can be used because the zone of maximum deformation concentration is of less importance. The problem with cutting velocity is that its effect on the process flow depends on the other two factors. Cutting velocity has the greatest influence on the burr height when used in the upper part of the range (*c_h_* > 0.06 mm). If clearances below this value are used, the velocity effect is smaller, but the rake angle *α* effect is increased, especially on the values of maximum cut edge deformations.

## 6. Conclusions

The main objective of the paper was an analysis of the shearing mechanism of the grain-oriented silicon steel cutting process. From the fundamental and practical point of view, knowledge about the mechanisms of forming the cut edge and its quality depending on the adopted criteria and conditions for the implementation of processes is crucial. The influence of the most important factors and technological parameters of the burr formation process and the mechanically affected zone was analyzed using experimental and numerical investigations. From the technological point of view, the reduction in damage zone and burr height is necessary to obtain workpieces with optimal magnetic properties without hysteresis loss and change the distribution of flux density. The results showed that by controlling the horizontal clearance, rake angle and shearing velocity parameters in a given case, it is possible to obtain a high-quality product. The following conclusions can be drawn from the conducted simulation and experimental studies:

- The developed simulations were prepared, taking into account the high accuracy of crack propagation. Such accuracy was obtained by introducing the variable of shape factor, which was *k* = 1, and the very fine mesh with a size of 0.015 mm.
- In the process of cutting magnetic materials, it is very important to reduce the degraded zone in the cutting edge to a minimum. The maximum equivalent plastic strain has reached a value of 1.9 for parameter configuration: *c_h_* = 0.04 mm, *α* = 10°, *v*_2_ = 13 m/min and it exponentially decays away from the edge. This parameter setting is inconvenient due to the concentration of large plastic strains on the cut edge.
- The most homogeneous surface of the cut edge with the minimum burr height was obtained for the following parameters: *α* = 15–30°, *c_h_* = 0.03 mm and *v*_2_ = 15 m/min. These conditions are the most favorable and can be used on production lines if the materials of identical thickness and similar mechanical properties are used.

## Figures and Tables

**Table 1:** Mechanical and magnetic properties of ET 122-30 steel (at *T* = 20 °C).

Density [kg/dm^3^]	Yield Point [MPa]	Elongation [%]	Hardness [HV_5_]	Maximum Specific Total Core Loss at 50 Hz at 1.7 T [W/kg]	Typical Magnetic Induction for H = 800 A/m [T]
7.65	300	11	160	1.22	1.87

**Table 2:** The ranges of the variability of the studied factors.

**Horizontal Clearance**, *c_h_*	0.02–0.15 mm
**Vertical Clearance**, *c_v_*	0.1 mm
**Slitting Velocity**, *v*_2_	3–24 m/min
**Rake Angle**, *α*	5–40°

## Data Availability

Data sharing is not applicable to this article.

## Nomenclature

*c_v_*	Vertical clearance	α	Rake angle
*c_h_*	Horizontal clearance	$\Delta \varepsilon_{ij}^{}$	Incremental component of the total strain tensor
$\Delta \varepsilon_{ij}^{\left(TE\right)}$	Incremental component of the thermo-elastic strain tensor	$\Delta \varepsilon_{ij}^{\left(VP\right)}$	Incremental component of the viscoplastic strain tensor
$\Delta \varepsilon_{ij}^{\left(TH\right)}$	Incremental component of the thermal strain tensor	$\Delta \sigma_{ij}^{}$	Incremental components of the second Piola-Kirchhoff stress
$\varepsilon_{kl}^{\left(E\right)}$	Accumulated components of the elastic strain tensor at time t	$\delta_{ij}$	Kronecker delta
E(T)	The temperature-dependent Young modulus	ν(T)	The temperature-dependent Poisson ratio
$\alpha_{m}$	Coefficient of thermal expansion	*v*_1_, *v*_2_	Slitting velocity
a, b, h	Finite element dimensions	*r*_1_, *r*_2_, *r*_3_	Radius tools
*w*_1_	Sheet width	*l*	Sheet length
*w*_1_, *w*_2_	The width of the knives	*F**_S_*, *F**_D_*	Static and dynamic friction coefficients
*D_C_*	Exponential decay coefficient	*v_rel_*	Relative velocity of the surfaces in contact
*σ_Y_*	Yield stress	*ε_eq_*	Equivalent plastic strain
${\dot{\varepsilon }}^{*}$	Normalized effective plastic strain rate	*m*	Thermal softening coefficient
*n*	Strain hardening coefficient	*T_m_*	Material melting temperature
*T_r_*	Room temperature	*k*	Shape factor
$\Delta \sigma_{eq{,}_{max}}$	Increment of the von Mises equivalent stress	*b_h_*	Burr height
